# Anti-inflammatory, antioxidant, antihypertensive, and antiarrhythmic effect of indole-3-carbinol, a phytochemical derived from cruciferous vegetables

**DOI:** 10.1016/j.heliyon.2022.e08989

**Published:** 2022-02-17

**Authors:** Natalia Jorgelina Prado, Daniela Ramirez, Luciana Mazzei, Micaela Parra, Mariana Casarotto, Juan Pablo Calvo, Darío Cuello carrión, Amira Zulma Ponce Zumino, Emiliano Raúl Diez, Alejandra Camargo, Walter Manucha

**Affiliations:** a Institute of Experimental Medicine and Biology of Cuyo (IMBECU)-UNCuyo-CONICET, Mendoza 5500, Argentina; b Facultad de Ciencias Médicas, Universidad Nacional de Cuyo, Mendoza 5500, Argentina; c Instituto de Biología Agrícola de Mendoza, (IBAM), Universidad Nacional de Cuyo, CONICET, Mendoza, Argentina; d Facultad de Ciencias Agrarias, Chacras de Coria, Mendoza, Argentina

**Keywords:** Indole-3-carbinol, Inflammation, Hypertension, Oxidative stress, Arrhythmias, Hsp70

## Abstract

**Background:**

Cardiovascular inflammation and oxidative stress are determining factors in high blood pressure and arrhythmias. Indole-3-carbinol is a cruciferous-derived phytochemical with potential anti-inflammatory and antioxidant effects. However, its implications on the modulation of cardiovascular inflammatory-oxidative markers are unknown.

**Objectives:**

To establish the effects of indole-3-carbinol on the oxidative-inflammatory-proarrhythmic conditions associated with hypertension.

**Materials:**

Histological, biochemical, molecular, and functional aspects were evaluated in 1) Culture of mouse BV-2 glial cells subjected to oxidative-inflammatory damage by lipopolysaccharides (100 ng/mL) in the presence or absence of 40 μM indole-3-carbinol (n = 5); 2) Male spontaneously hypertensive rats (SHR) and Wistar Kyoto rats receiving indole-3-carbinol (2000 ppm/day, orally) during the first 8 weeks of life (n = 15); 3) Isolated rat hearts were submitted to 10 min regional ischemia and 10 min reperfusion.

**Results:**

1) lipopolysaccharides induced oxidative stress and increased inflammatory markers; indole-3-carbinol reversed both conditions (interleukin 6, tumor necrosis factor α, the activity of nicotinamide adenine dinucleotide phosphate oxidase, nitric oxide, inducible nitric oxide synthase, heat shock protein 70, all p < 0.01 vs lipopolysaccharides). 2) SHR rats showed histological, structural, and functional changes with increasing systolic blood pressure (154 ± 8 mmHg vs. 122 ± 7 mmHg in Wistar Kyoto rats, p < 0.01); Inflammatory-oxidative markers also increased, and nitric oxide and heat shock protein 70 decreased. Conversely, indole-3-carbinol reduced oxidative-inflammatory markers and systolic blood pressure (133 ± 8 mmHg, p < 0.01 vs. SHR). 3) indole-3-carbinol reduced reperfusion arrhythmias from 8/10 in SHR to 0/10 (p = 0.0007 by Fisher's exact test).

**Conclusions:**

Indole-3-carbinol reduces the inflammatory-oxidative-proarrhythmic process of hypertension. The nitric oxide and heat shock protein 70 are relevant mechanisms of indole-3-carbinol protective actions. Further studies with this pleiotropic phytochemical as a promising cardioprotective are guaranteed.

## Highlights

• Indole-3-carbinol, a cruciferous-derived compound, has cardioprotective potential.
• We confirmed its anti-inflammatory and antioxidant effects *in vitro* and *in vivo.*
• Oral administration reduced blood pressure and cardiac remodeling.
• In isolated hearts from hypertensive rats prevented ischemia-reperfusion arrhythmias.
• Heat shock protein 70 and NO contribute to indole-3-carbinol protective actions.

## Graphical abstract

**Figure:**
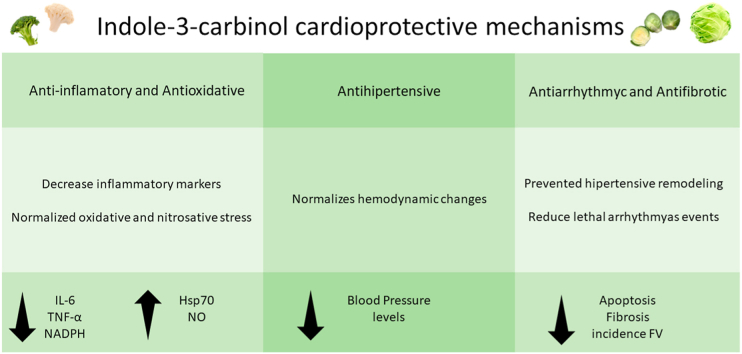

## 1 Introduction

Cardiovascular disease contributes significantly to morbidity and mortality throughout the world. Inflammation and oxidative damage are critical factors in cardiovascular pathogenesis. In this sense, alterations in molecular mediators such as nitric oxide (NO) and heat shock protein 70 (Hsp70) would determine remodeling changes associated with cardiovascular disease, especially during arterial hypertension. A diet low in phytochemical content contributes to cardiovascular risk because its deficit favors inflammation and oxidative stress (reducing NO levels) and, consequently, increases atherosclerosis, endothelial dysfunction, and vascular inflammation.

Higher NO bioavailability induces Hsp70 expression and boosts antioxidant defenses. This mechanism would participate directly in protecting the cardiovascular system. Recent research suggests this signaling pathway as a valid hypothesis that justifies the use of so-called functional foods as a strategy to prevent vascular aging and the development of atherosclerosis (Kitada et al., 2016).

Functional foods stand out for their high content of active compounds (phytochemicals) and show beneficial properties in relation to health (Camargo and Manucha, 2017). One of these compounds is indole-3-carbinol (I3C), which is found in vegetables belonging to the cruciferous genus, such as broccoli and cabbage (Licznerska and Baer-Dubowska, 2016). This phytochemical demonstrated cardioprotective, antioxidant, and anti-inflammatory effects against the cardiotoxicity of doxorubicin (Adwas et al., 2016; Hajra et al., 2018; Liu et al., 2020).

Furthermore, a recent finding reported that I3C decreases the inflammatory response produced by the ischemia-reperfusion model (Ampofo et al., 2017). Therefore, the authors suggest that the use of foods containing I3C could benefit the treatment of cardiovascular inflammation due to ischemia-reperfusion. However, it has not been elucidated whether such treatment would reduce the fatal arrhythmias typical of post-ischemic reperfusion.

Arrhythmias are the leading cause of cardiovascular mortality worldwide. Most deaths in acute coronary syndromes are due to ventricular arrhythmias within the first hour of the onset of symptoms. This is due to the interaction of various chemicals present in reperfused myocardial tissue, including cAMP, catecholamines, and oxygen-derived free radicals, all of which are arrhythmogenic factors. On the other side, several studies show that NO functions as an endogenous mediator of cardioprotection in the isolated rat heart through the ischemia-reperfusion model, reducing the incidence of arrhythmias (Pabla and Curtis, 1995; Prado et al., 2018; Enayati et al., 2018).

Based on the exposed above, we aim to evaluate I3C effect on the oxidative-inflammatory-proarrhythmic conditions associated with hypertension. The acute anti-inflammatory and antioxidant effects with special attention on Hsp70 and NO production were evaluated, in BV-2 cells -a recognized cell line used for inflammatory/oxidative assays-. The cardioprotective potential of a chronic I3C treatment against hypertensive myocardial remodeling and reperfusion arrhythmias were evaluated in SHR.

## 2 Materials and methods

### 2.1 Cell culture and treatment

The BV-2 cells were cultured and treated according to the specifications of a previous report (Mazzei et al., 2020). Specifically, treatments were started after one day of quiescence (0.1% FBS). Cells were damaged or not with lipopolysaccharides (LPS, 100 ng/mL), and simultaneously co-treated or not with 40 μM I3C (Kim et al., 2014). I3C (>96% v/v) was obtained from Sigma Aldrich Co. (St. Louis, MO, USA). Each protocol (triplicate) was replicated five to ten times. The treatments lasted 72 h, and then the cells were harvested to carry out the planned determinations.

#### 2.1.1 Measure for cellular oxidative stress

We determined the superoxide anion production by the dihydroethidium fluorophore (DHE; Invitrogen, Grand Island, NY, USA) according to previous publications (Martínez-Martínez et al., 2016; Mazzei et al., 2020). Briefly, BV-2 cells were incubated with DHE (5 × 10^−3^ mmol/L) for 30 min in a light-protected humidified chamber at 37 °C. Cells were subsequently washed with warm PBS and analyzed with a 40X objective in a fluorescent laser scanning Leica DMI 3000 microscope (Ex561 nm and Em610 nm). For each condition, approximately 200 cells were analyzed. The mean fluorescence densities in the nucleus were calculated. A researcher unaware of the experimental groups performed the analysis. The levels of the superoxide anion are presented as an n-fold increase over the values of the control group. These correspond to five assays, and each one was done in triplicate.

#### 2.1.2 Determination of nitrite levels in BV-2 cells

The Griess reaction was used to determine nitrites (nitric oxide indirectly). Cellular homogenates were incubated with 10 mmo^1^/L l-Arginine in a buffer (pH 7.40) containing 25 mmol/L HEPES, 140 mmol/L NaCl, 5.4 mmol/L KCl, 1.8 mmol/L CaCl_2_, 1 mmol/L MgCl_2_, and 5 mmol/L glucose at 37 °C for 24 h. After centrifugation at 6,400 rpm for 20 min, the supernatants were used for the assay of indirect NO production and the amount was corrected by means of the protein amount, measured according to the Bradford method. Nitrites (nmol/μg protein/100 μL homogenate) were determined by spectrophotometry at 540 nm (Mazzei et al., 2010, 2020).

#### 2.1.3 Mitochondrial isolation technique

Mitochondrial extraction from BV-2 cells was carried out according to a previous report (Prado et al., 2018), and then, we verified the purity of the mitochondrial fraction.

#### 2.1.4 NADPH activity assay

Luminol technique (5-amino-2,3-dihydro-1,4-phthalazine, Sigma-Aldrich) was used to determine the NADPH oxidase activity in BV-2 cells, as well as in their mitochondrial fractions. The samples were processed using methodologies, reagents and equipment such as those used in previous reports from our laboratory (García et al., 2012; Prado et al., 2018). Final results are shown as arbitrary fluorescence units (AFU)/microgram of protein/minute of incubation.

#### 2.1.5 Protein detection and quantification by Western blot technique

According to previously reported (Manucha and Vallés, 2008), the protein levels were determined. The specific primary antibodies used were: IL-6 (ab229381 rabbit monoclonal, 1:1500), TNF-α (ab215188 rabbit monoclonal, 1:1500), p22-phox (ab75941 rabbit polyclonal, 1:1000), iNOS (sc-7271 mouse monoclonal, 1:2500), and Hsp70 (sc-33575 rabbit polyclonal, 1:1000). While for the expression of housekeeping genes, we use an anti-β-actin (sc-47778 mouse monoclonal antibody, 1:2000) for cells and an anti-COX IV (ab-16056 rabbit polyclonal antibody, 1:1500) for mitochondrial fractions. The ratios of the immunosignal were standardized to 100 for the corresponding control values (BV-2 cells without treatment).

#### 2.1.6 Immunofluorescence confocal microscopy

All the methodologies, equipment, primary and secondary antibodies used were following previous reports and for more details see (García et al., 2012, 2014; Prado et al., 2018). Primary antibodies: monoclonal anti-TNF-α (1:100) (Abcam) and monoclonal anti-Tubulin (1:100) (Santa Cruz Biotechnology). Secondary antibodies were goat anti-rabbit IgG antibody, Cy2 conjugated (1:750) and donkey anti-mouse IgG antibody, Cy3 conjugated (1:750). After being washed, the tissues were stained with DAPI, hydrochloride (Thermo Fisher Scientific, 10 nM) for 5 min.

### 2.2 In vivo protocols

The protocol agreed with the regulations of the Institutional Committee for the Care and Use of Laboratory Animals of the Faculty of Medical Sciences of the UNCuyo (Protocol approval: 153/2019). We perform the study in male spontaneously hypertensive rats (SHR) and Wistar Kyoto rats (WKY), that received or not I3C (2,000 ppm/day, *per os* by gavage) from birth to 8 weeks of life (n = 15, for each group). I3C was prepared in corn oil before use.

#### 2.2.1 Systolic blood pressure

Since SHR develop a clear increase in blood pressure from the 5th or 6th week of life, we measured blood pressure at weeks 6th and 8th. Systolic blood pressure was measured using the CODA® tail-cuff blood pressure system (Kent Scientific Corporation) (Daugherty et al., 2009; Martín Giménez et al., 2019).

#### 2.2.2 Histology

After the 8 weeks, five cardiac samples were collected for histology (Masson's trichrome, histochemistry, and TUNEL) as previously described (Prado et al., 2018). For the quantification of apoptotic cells in cross-sectioned areas, a total of ten consecutive fields were randomly selected and were evaluated using an image analyzer.

Morphological evaluations were performed in the same way as in a previous report (Diez et al., 2015). The collagen interstitial was also estimated according to the procedures described previously (Diez et al., 2015; Prado et al., 2018).

The Hsp70 immunohistochemical studies were performed according to García et al. (2014). In brief, the sections were immunostained to reveal Hsp70. Antibody applied was rabbit polyclonal antibody against Hsp70 (H-300) diluted at 1:500. A commercial immunoperoxidase kit was used (Dako Corporation, Carpinteria, CA, USA). The immunostaining was evaluated and resolved by consensus according to a scoring system as the following intensity scores: 0, 1, 2, and 3 as no staining, weak staining, moderate staining, and strong staining, respectively Prado et al. (2018).

#### 2.2.3 RT-PCR and Western blot techniques

Also, cardiac tissues were separated for molecular assays as previous report (Manucha and Vallés, 2008). Subsequently, ribonucleic acid was obtained from the ventricular tissue, denatured and subjected to the reverse transcription technique as previously described (Diez et al., 2015; Prado et al., 2018). Each sample was analyzed to determine levels of IL-6, TNF-α, iNOS, Hsp70, and β-actin. Set primers used: IL-6 Sense 5′-CAAGAGACTTCCAGCCAGTTGC-3′, Antisense 5′-CAAGAGACTTCCAGCCAGTTGC-3′; TNF-α Sense 5′-AAGCCTGTAGCCCACGTCGTA-3′, Antisense 5′- GGCACCACTAGTTGGTTGTCTTTG-3´; iNOS Sense 5′-GCATGGACCAGTATAAGGCAAGCA-3′, Antisense 5´--GCTTCTGGTCGATGTCATGAGCAA-3´; Hsp70 Sense 5′-CCGCCTACTTCAACGACTC-3′, Antisense 5′-TCTTGAACTCCTCCACGAAG-3′, and β-Actin Sense 5′-TGGAGAAGAGCTATGAGCTGCCTG-3′, Antisense 5′-GTGCCACCAGACAGCACTGTGTTG-3´.

In parallel, ventricular myocardial tissues were homogenized for Western blot assay according to Manucha and Vallés (2008). The antibodies, conditions, and trademarks were the same as those used in the protocol with BV-2 cells. Densitometric analysis was carried out by image analysis software; the photographs were digitalized by using a scanner (LACIE Silver Scanner for Macintosh) and the Desk Scan software (Adobe Photoshop). The ratios of the immunosignal were standardized to 100 for the corresponding control values (WKY).

#### 2.2.4 Ischemia/reperfusion arrhythmias

Ten isolated hearts were used for the ischemia/reperfusion protocol. Hearts were perfused as previous reports (Diez et al., 2015; Prado et al., 2018). In WKY hearts' the perfusion pressure was kept constant at 80 cm H_2_0, whereas, in the SHR, it was raised to 120 cm H_2_0. The stabilization protocol, pre-ischemia and reperfusion, was also performed according to Diez et al., 2015 as well Prado et al., 2018. Finally, the arrhythmias incidence and severity quantification followed the Lambeth convention and a score previously described (Curtis et al., 2013; Diez et al., 2013; Prado et al., 2018).

### 2.3 Statistical analysis

Data were expressed as mean ± S.D. and processed by ANOVA and Bonferroni post-test for most of the comparison in cell cultures and animal studies. Repeated measures ANOVA II and Bonferroni post-test were used to analyze systolic blood pressure and the arrhythmia severity score during ischemia and reperfusion. The sample size required to detect a 50% reduction in reperfusion arrhythmias incidence is 10 animals per group. The incidence was compared using Fisher's exact test. The Prism software for Windows (version 9.2.0, GraphPad Software, San Diego, CA, USA) was used for all statistical analyses. A significance level of p < 0.05 was established.

## 3 Results

### 3.1 Anti-inflammatory and antioxidant actions of I3C in BV-2 cultures exposed to LPS

**Figure 1:**
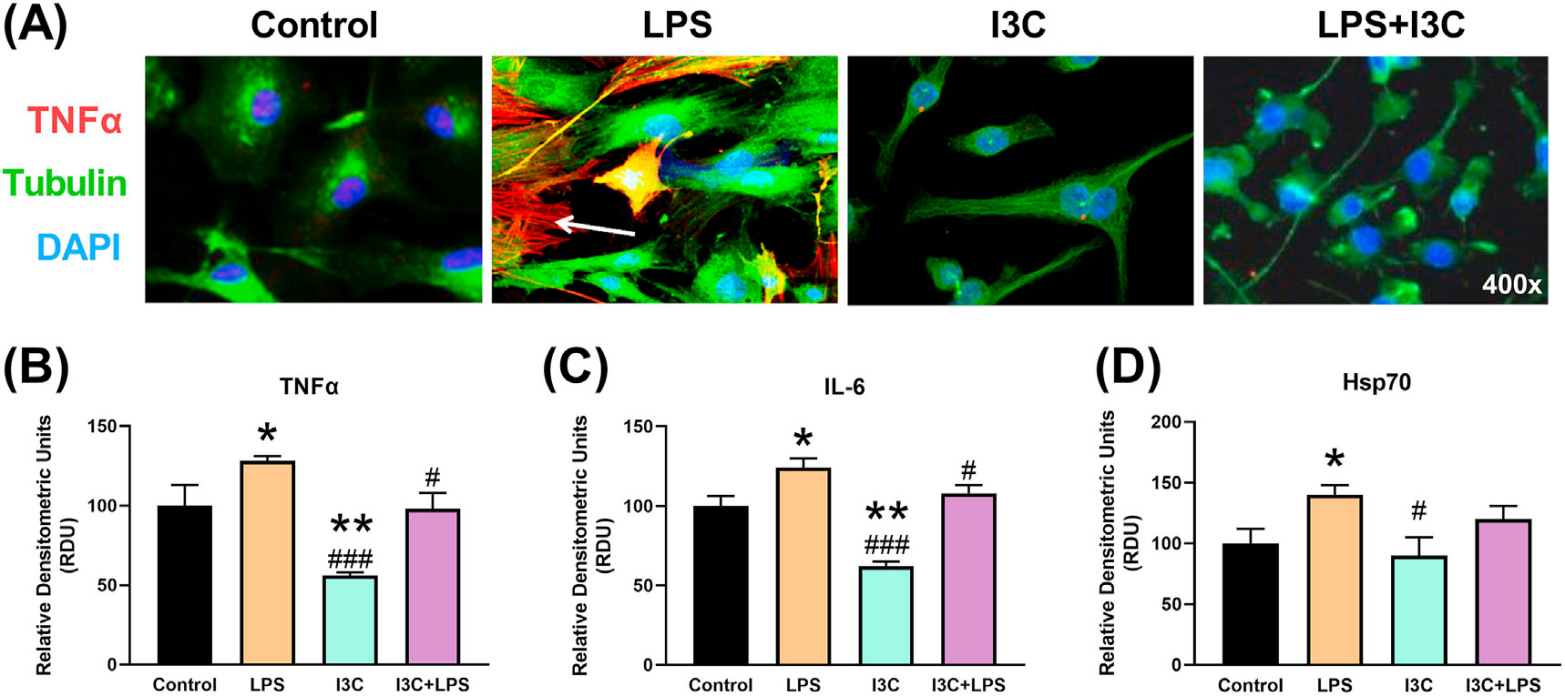
Indole-3-carbinol (I3C) prevented lipopolysaccharides-induced inflammation in mouse Bv-2 cells. (A) Representative confocal images confirmed the increase in tumor necrosis factor α (TNFα) induced by lipopolysaccharides (LPS) and the anti-inflammatory effect of I3C. (B) Levels of TNFα, (C) interleukin-6 (IL-6), and (D) heat shock protein 70 (Hsp70) by RT-PCR. ∗p < 0.05 vs control and #p < 0.05, ##p < 0.01, ###p < 0.001 vs LPS.

An increase in the levels of TNF-α and IL-6 confirmed an inflammatory response to LPS in mouse BV-2 cells (Figure 1). The Hsp70 responded to inflammatory stress with increased expression. I3C reduced the inflammatory markers.

**Figure 2:**
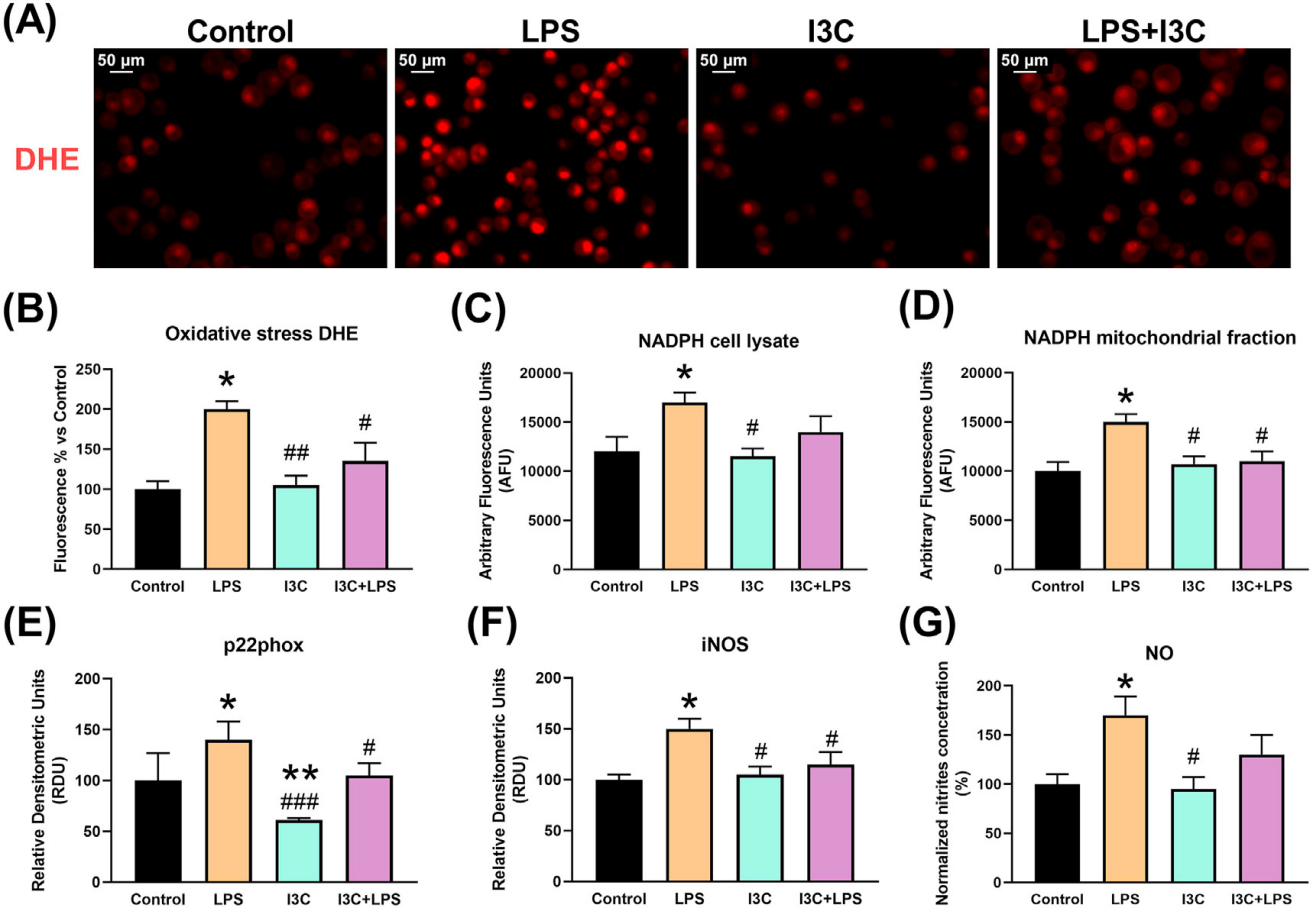
I3C prevented LPS-induced oxidative stress in mouse Bv-2 cells. (A) I3C reduced superoxide anion damage measured by dihydroethidium (DHE) fluorescence. (B) The quantitation confirmed the antioxidant protection by I3C. (C) NADPH activity was enhanced by LPS and effectively attenuated in the mitochondrial fractions (D) by I3C Oxidative stress markers induced by LPS. (E) The p22phox subunit of NADPH augments its levels in the presence of LPS, but this is neutralized in the presence of I3C. (F) LPS increased inducible nitric oxide synthase (iNOS) enzyme expression regardless of LPS stimulus and (G) NO levels increased in the stressed cells. I3C did not reduced NO levels. ∗p < 0.05 vs control and #p < 0.05, ##p < 0.01, ###p < 0.001 vs LPS.

As shown in Figure 2, oxidative stress measured through DHE, NADPH activity in cell lysates and mitochondrial fractions, and NADPH p22phox subunit expression in the LPS cell model, returned to control levels with the administration of I3C. The Hsp70 and iNOS respond to inflammatory stress with increased expression and the phytochemical limited both. I3C limited NO overproduction.

### 3.2 I3C prevented hypertension-induced cardiac remodeling

**Figure 3:**
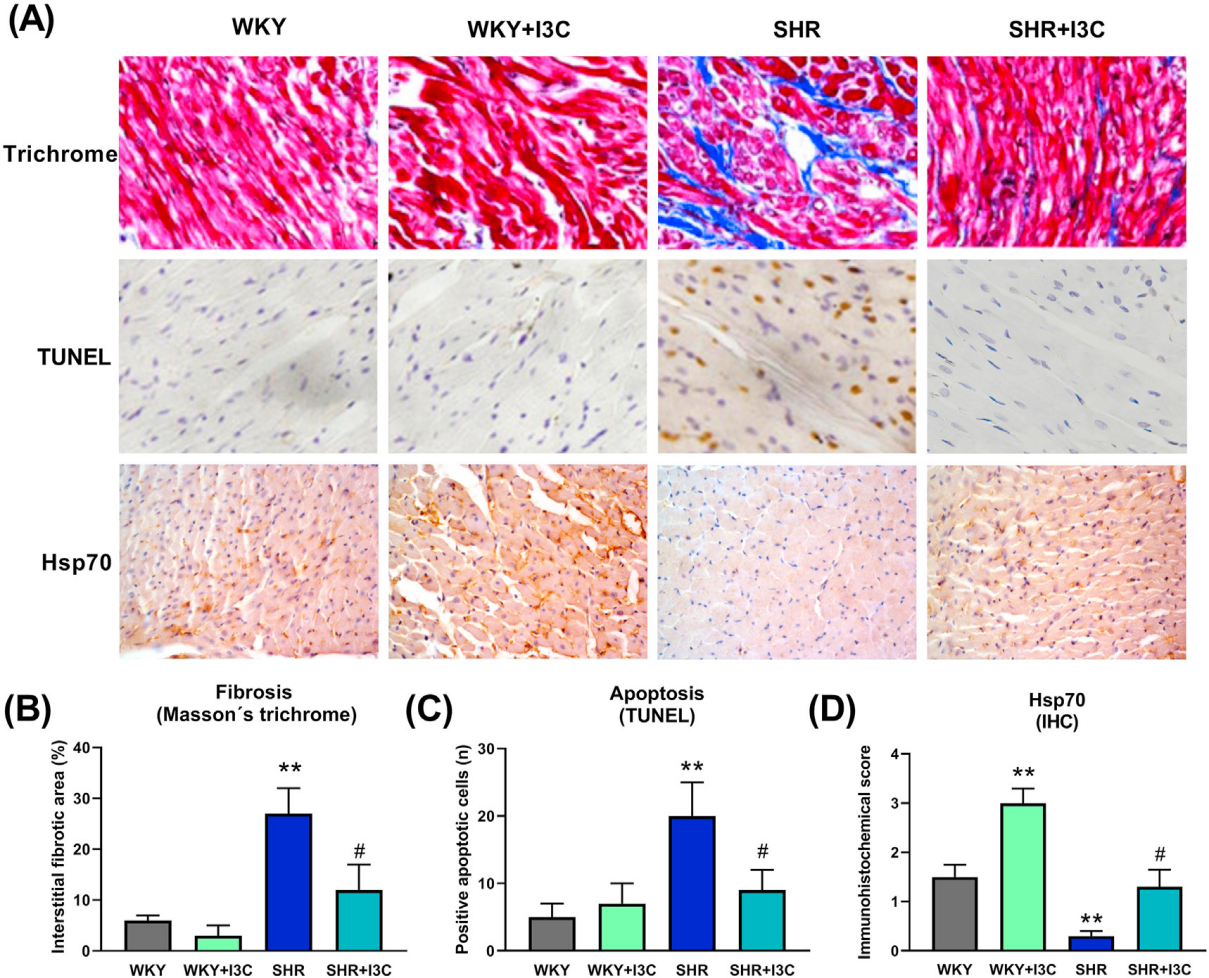
I3C prevented hypertensive remodeling. (A) Representative images of increased fibrosis and apoptosis, and decreased Hsp70 in hearts from spontaneously hypertensive rats (SHR) and the preventive effect of chronic oral I3C administration. (B) Quantitation of Masson's trichrome and (C) TUNEL staining confirmed the antiremodeling action of I3C, (D) and the increased levels of Hsp70 in both normotensive (WKY) and hypertensive rat hearts. ∗∗p < 0.01 vs WKY and #p < 0.05 vs SHR.

Hears of the SHR rats presented an increase in fibrosis and apoptosis, and conversely a decrease in Hsp70 expression (Figure 3). Apoptosis and fibrosis were significantly reduced by the phytochemical. Interestingly, the levels of Hsp70 were higher in the normotensive rats that received I3C.

### 3.3 Anti-inflammatory, antioxidant, and antihypertensive effect of the I3C in SHR

**Figure 4:**
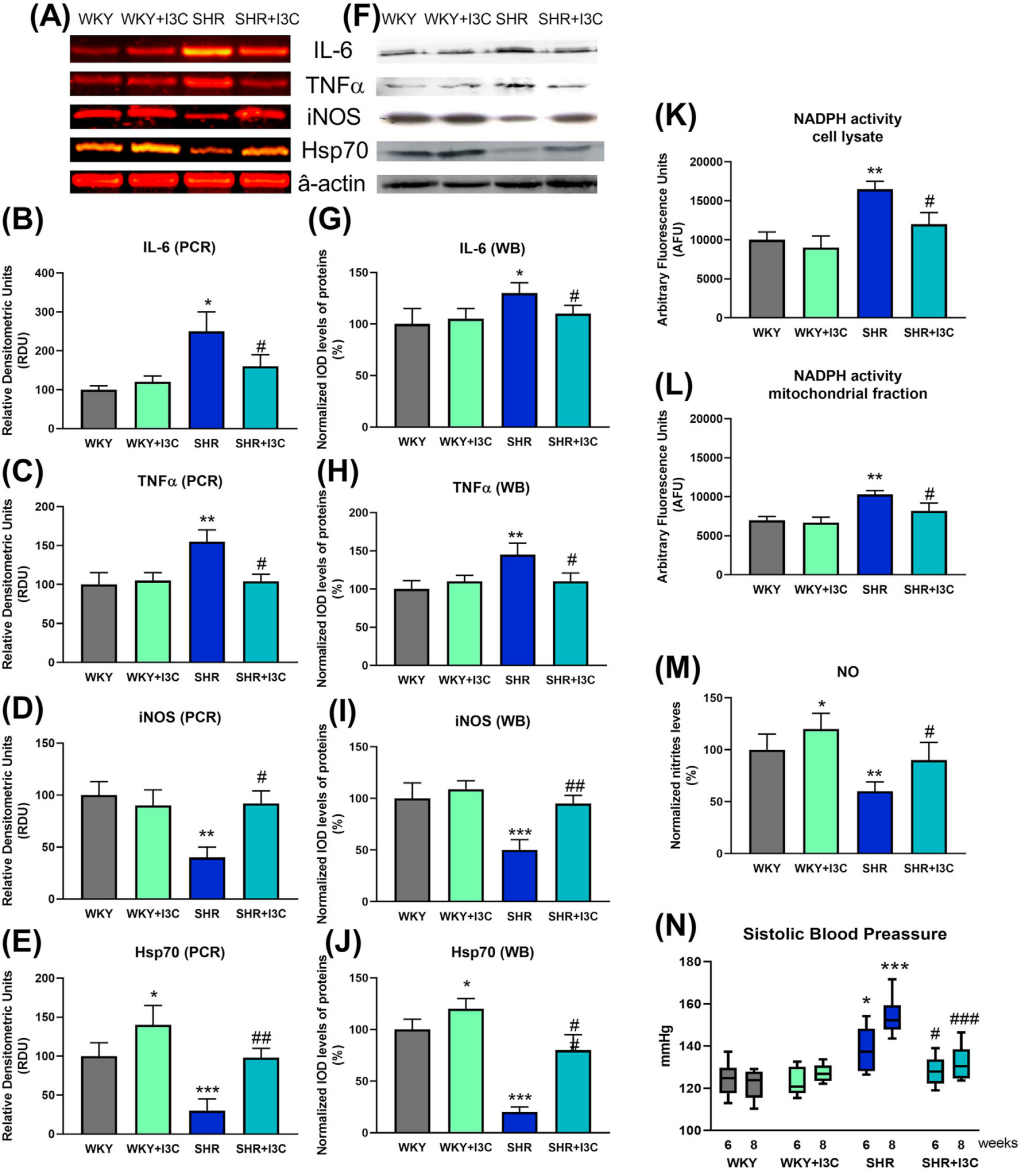
I3C reduced inflammatory and oxidative stress in SHR rat hearts. (A) Representative results of IL-6, TNFα, iNOS, Hsp70 and their housekeeper β-actin of RT-PCR and (F) Western blot for the hearts of normotensive and hypertensive rats treated or not with I3C. (B) the mRNA of IL-6 and (C) TNFα increased and (D) iNOS and (E) Hsp70 decreased in the SHR group and I3C restored the levels of these markers to those found in WKY. (G, H, I, J) these results were confirmed in the quantitated analysis of the protein expression. (K) NADPH activity increased in cell lysates and (L) mitochondrial fractions from SHR hearts, and I3C prevented the increase in both types of samples. (M) I3C augmented NO availability in WKY and prevented the decrease in SHR. (N) I3C prevented the increase in blood pressure in spontaneously hypertensive rats at 6 and 8 weeks of life. ∗p < 0.05, ∗∗p < 0.01, ∗∗∗p < 0.001 vs WKY; and #p < 0.05, ##p < 0.01, ###p < 0.001 vs SHR.

The mRNA and the protein levels of IL-6 and TNF-α increased in SHR rats (Figure 4). I3C reduced both inflammatory markers. The phytochemical increased Hsp70 in normotensive rats and prevented the reduction observed in SHR rats. I3C prevented the increase in the activity of the NADPH in cell lysate and mitochondrial fractions of SHR. The levels of iNOS and NO production were restored in samples from SHR treated with I3C. At week 6 the SHR was the only group with high systolic blood pressure (Figure 4N). I3C demonstrated antihypertensive potency at week 8 by reducing blood pressure values in SHR rats 21.2 mmHg (95% confidence interval 12.9–29.6 mmHg, p < 0.001).

### 3.4 Antiarrhythmic protection of I3C in hearts from SHR submitted to ischemia/reperfusion

**Figure 5:**
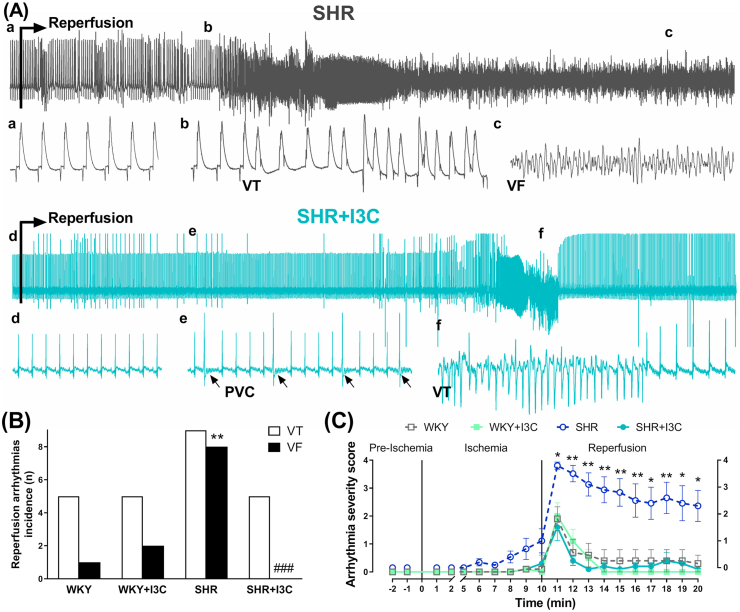
Antiarrhythmic action of I3C. (A) Representative ECG traces of the last 2 s of ischemia and the first 2 min of reperfusion. Lower case letters indicate the beginning of corresponding trace in an augmented time scale. Samples of ventricular tachycardia (VT) and ventricular fibrillation (VF) are indicated and premature ventricular complex (PCV) pointed by black arrows (B) VT and VF in white and black columns quantified a proarrhythmic response in SHR hearts and a marked anti-fibrillatory effect of I3C. (C) The evolution and severity of arrhythmias during the ischemia/reperfusion protocol presented a persistent increase in the SHR group that was prevented by I3C. ∗p < 0.05, ∗∗p < 0.01 vs WKY; and ###p < 0.001 vs SHR.

Ventricular tachycardia occurred in all groups during reperfusion (Figure 5). Hearts from SHR rats suffered a higher incidence of ventricular fibrillation that started in the first minute of reperfusion, and in most of the cases persisted fibrillated during the 10 min of the reperfusion period. The hearts from WKY, WKY + I3C and SHR + I3C groups display a similar electrophysiological response, with brief arrhythmic episodes mainly during the first couple of minutes of reperfusion.

## 4 Discussion

This work describes the cardioprotective action of I3C, a promising phytochemical found in cruciferous plants. We confirm the anti-inflammatory and antioxidant effects of this compound in cultured BV-2 cells exposed to LPS and then replicate these protective properties in hypertensive rats. Furthermore, chronic oral administration of I3C prevented hypertensive remodeling and made the heart more resistant to reperfusion-induced arrhythmias.

To highlight, the anti-inflammatory and antioxidant effects of this phytochemical compound in cultured BV-2 cells exposed to LPS are consistent and reinforce a recent report (Khan and Langmann, 2020). Moreover, these findings on LPS assure that Hsp70 overexpression mitigates oxidative stress and inflammatory status (Mazzei et al., 2020). Our results regarding reducing inflammatory markers concur with those previously described in Raw and THP-1 cell lines (Chen et al., 2010; Jiang et al., 2013). However, Kim et al. (2014) did not find an anti-inflammatory effect of I3C in BV-2 glial cells, but in its metabolite, diindolylmethane (DIM). The treatment used in the present work -100 ng/mL of LPS during 72 h- is in accord with the same dose implemented by Kim et al. (2014), but they exposed the cells only during 12h. The I3C dose we used to match the higher reported that did not induce cellular toxicity. A time-dependent factor could be involved in the different effects observed. In 8 different cell cultures, more than 50% of I3C transforms into DIM in 24 h by spontaneous dimerization. At 48 h, greater than 60% conversion was reported (Brandlow and Zeligs, 2010). However, I3C and DIM suppress LPS-stimulated inflammatory responses in murine macrophages (Cho et al., 2008; Tsai et al., 2010). We cannot rule out the high probability that I3C transformed to DIM, both *in vitro* and *in vivo*, because we did not measure DIM levels; therefore, we attribute the effect to I3C even if it may be acting as a pro-drug. We found that I3C prevented the induction of iNOS by LPS. In the context of cell culture, this can be considered a cellular stress response, whereas the increment in vascular tissue can lead to an improvement in NO availability and consequent vasodilation. The above results clearly correspond with the antihypertensive action found in this work.

We described anti-inflammatory and antioxidant actions of I3C for the first time in SHR rats. Previous reports of I3C in relation to hypertension were made using a transgenic rat model with I3C as a mouse renin transgene inducer (cyp1a1ren-2 transgenic rats) (Kantachuvesiri et al., 2001; Mitchell et al., 2006; Prieto et al., 2011). The induction of Cyp1a1 promoter is mediated by oral I3C, which drives the expression of the Ren2 renin gene in the liver and grades the increase in blood pressure in a dose-dependent manner. Interestingly, the effects of I3C in the male F344 rats, the normotensive controls of cyp1a1ren-2 transgenic rats, showed a reduction in mRNA endothelin-1 and the NOX-2 isoform of NADP oxidase (Peters et al., 2009). These results are in agreement with the antioxidant effects and with the prevention of myocardial remodeling described here. Furthermore, I3C presents antifibrotic properties in a mice model of heart failure that were lost in AMPKα2 protein KO mice (Deng et al., 2014). Our results reaffirm the antihypertensive and antifibrotic profile of this phytochemical.

I3C is a potential cardioprotective agent because it prevents oxidative and nitro-oxidative damage, inflammation, and apoptosis in cardiac tissues exposed to doxorubicin or ischemia/reperfusion. Doxorubicin (DOX) is an anthracycline used for cancer treatment that leads to cardiomyopathy and congestive heart failure. Our results agree with the protection against DOX-induced cardiotoxicity by NADPH modulation. Still, the activation of other antioxidant pathways can contribute to cardioprotection (heme oxygenase 1, quinine oxidoreductase 1, and glutathione reductase) (Adwas et al., 2016; Liu et al., 2020). I3C significantly attenuated DOX-induced inflammation by downregulation of pro-inflammatory mediators, NF-kβ, COX-2, IL-6, and iNOS expression (Hajra et al., 2018; Liu et al., 2020). Ampofo et al. (2017) reported that I3C reduces the ischemia/reperfusion-induced translocation of the NF-kB subunit p65, decreasing E-selectin and ICAM-1 expression on endothelial cells and suppresses the protein levels of Mac-1 and the activation of LFA-1 on leukocytes. The actions of I3C on endothelial and leucocytes reduce the transmigration into the post-ischemic tissue.

We highlight a novel cardioprotective action of I3C against reperfusion-induced arrhythmias. The prevention of hypertensive-related remodeling could be the critical determinant of I3C antiarrhythmic action. Additionally, the increase in Hsp70 and its anti-inflammatory and antioxidant effects can also contribute to the marked reduction of ventricular fibrillation, the most severe and lethal type of arrhythmia (see Figure 4). Additional studies will be needed to explore the antiarrhythmic mechanisms of this phytochemical.

## 5 Conclusion

We conclude that I3C is a promising cardioprotective phytochemical. We highlight the key role of increasing Hsp70 as a mechanism that combines anti-inflammatory and antioxidant in the protective effects of a compound found in cruciferous vegetables. The preventive effects against hypertension-induced cardiac remodeling and arrhythmias guarantee further studies from a pharmacological perspective as well as validation as cardioprotective functional food.

## Declarations

### Author contribution statement

Natalia Jorgelina Prado: Performed the experiments; Wrote the paper.

Daniela Ramirez, Luciana Mazzei, Micaela Parra, Mariana Casarotto, Juan Pablo Calvo: Performed the experiments; Analyzed and interpreted the data.

Darío Cuello CarriÓN: Performed the experiments; Contributed reagents, materials, analysis tools or data.

Amira Zulma Ponce Zumino, Emiliano Raúl Diez, Alejandra Camargo, Walter Manucha: Conceived and designed the experiments; Analyzed and interpreted the data; Wrote the paper.

### Funding statement

This work was supported by the Research and Technology Council of Cuyo University (SECyT), Mendoza, Argentina, and the National Agency for the Promotion of Research, Technological Development and Innovation ANPCyT FONCyT (PICT 2016-4541).

### Data availability statement

Data included in article/supplementary material/referenced in article.

### Declaration of interests statement

The authors declare no conflict of interest.

### Additional information

No additional information is available for this paper.
